# Mycoremediation of Flotation Tailings with *Agaricus bisporus*

**DOI:** 10.3390/jof8080883

**Published:** 2022-08-22

**Authors:** Sylwia Budzyńska, Marek Siwulski, Anna Budka, Pavel Kalač, Przemysław Niedzielski, Monika Gąsecka, Mirosław Mleczek

**Affiliations:** 1 Department of Chemistry, Poznan University of Life Sciences, Wojska Polskiego 75, 60-625 Poznań, Poland; 2 Department of Vegetable Crops, Poznan University of Life Sciences, Dąbrowskiego 159, 60-594 Poznań, Poland; 3 Department of Mathematical and Statistical Methods, Poznan University of Life Sciences, Wojska Polskiego 28, 60-637 Poznań, Poland; 4 Department of Applied Chemistry, Faculty of Agriculture, University of South Bohemia, 370 04 České Budějovice, Czech Republic; 5 Faculty of Chemistry, Adam Mickiewicz University in Poznań, Uniwersytetu Poznańskiego 8, 61-614 Poznań, Poland

**Keywords:** accumulation, basidiomycete, bioremediation, champignon, common mushroom, edible mushroom, sludge, toxic elements, wastes

## Abstract

Due to their enzymatic and bioaccumulation faculties the use of macromycetes for the decontamination of polluted matrices seems reasonable for bioremediation. For this reason, the aim of our study was to evaluate the mycoremediation ability of *Agaricus bisporus* cultivated on compost mixed with flotation tailings in different quantities (1, 5, 10, 15, and 20% addition). The biomass of the fruit bodies and the content of 51 major and trace elements were determined. Cultivation of *A. bisporus* in compost moderately polluted with flotation tailings yielded significantly lower (the first flush) and higher (the second flush) biomass of fruit bodies, compared with the control treatment. The presence of toxic trace elements did not cause any visible adverse symptoms for *A. bisporus*. Increasing the addition of flotation tailings to the compost induced an elevated level of most determined elements. A significant increase in rare earth elements (both flushes) and platinum group elements (first flush only) was observed. The opposite situation was recorded for major essential elements, except for Na and Mg in *A. bisporus* from the second flush under the most enriched compost (20%). Nevertheless, calculated bioaccumulation factor values showed a selective accumulation capacity—limited for toxic elements (except for Ag, As, and Cd) and the effective accumulation of B, Cu, K, and Se. The obtained results confirmed that *A. bisporus* can be used for practical application in mycoremediation in the industry although this must be preceded by larger-scale tests. This application seems to be the most favorable for media contaminated with selected elements, whose absorption by fruiting bodies is the most efficient.

## 1. Introduction

Mycoremediation as a form of bioremediation may be an effective, eco-friendly technique for decontamination of polluted environmental matrices because of its simplicity and highly efficient implementation process [1,2,3,4,5,6,7]. It is also one of the least costly forms of remediation, and both micromycetes and macromycetes may be used [8,9]. Fungi-mediated remediation as a cost-effective method may use mycelium to effectively secrete extracellular enzymes, finally transforming organic pollutants into non-toxic compounds (bioaugmentation) or accumulating toxic elements [10,11,12].

There are numerous literature data about biodegradation, bioconversion, or biosorption for the degradation of common pollutants using different mushroom species [13,14]. Biodegradation of polycyclic aromatic hydrocarbons by *Phanerochaete chrysosporium*, *Trametes versicolor*, *Pleurotus ostreatus,* and *P. eryngii*; pesticides and herbicides by *Botryosphaeria laricina*, *Aspergillus glaucus*, *T. pavonia*, *Penicillium spiculisporus,* and *P. verruculosum*; antibiotics and pharmaceuticals by *Leptospaherulina* sp, *Irpex lacteus*, *Lentinula edodes*, *Mucor hiemalis,* and *Phanerochaete chrysosporium* were reviewed by Akhtar and Mannan [9]. Effective biosorption of heavy metals (aluminium (Al), chromium (Cr), cobalt (Co), copper (Cu), lead (Pb), iron (Fe), nickel (Ni), manganese (Mn), and zinc (Zn)) with the use of spent *Agaricus bisporus* from production has also been described in the literature [4,15,16]. In terms of heavy metals, mycoremediation by easily cultivable, fast-growing, and highly accumulating white rot fungi *P. sajor-caju*, *A. bitorquis*, and *Ganoderma lucidum* with a potential for Cr, Cu, and Pb remediation was described by Hanif and Bhatti [17]. This method may also use macromycetes to accumulate toxic elements in their biomass [18]. An example of such a species is *Pleurotus* spp., which is known to effectively accumulate selected trace elements in whole fruit bodies [19]. This method limits the initial toxicity of elements after their accumulation without the risk of the production of toxic metabolites, which are usually present in bioremediation with microbes [20].

It should be remembered that despite many advantages, biological methods of environmental remediation also have limiting factors. In the review of Boopathy [20], various factors that limit the use of bioremediation technologies were summarized. Some information can be found in the literature on the critical aspects and limitations of the use of mycoremediation. They should not be forgotten and strategies to overcome them are necessary [9]. One of the examples of limiting factors is the reduced bioavailability of pollutants. Puglisi et al. [21] observed that some fungi overcome this limitation by the production of unique proteins (hydrophobins), due to their ability to dissolve hydrophobic molecules into aqueous media. For an effective process, optimal conditions should be present (temperature: 10–28 °C, pH: about 6.5, humidity: 86–90%, and CO_2_ level: 15–20%) [22,23]. In the case of the in situ process, maintaining the above-mentioned conditions can be difficult, which is another limitation. Rubichaud et al. [24] confirmed that cold environmental conditions can impede the activity of various fungal enzymes necessary to degrade toxic pollutants. Therefore, the selection of suitable macrofungi for the particular substrate is essential. A high rate of element accumulation combined with a more frequent harvest cycle is a clear argument for using this method in practice [25]. 

A separate issue is the efficiency of metal accumulation by fruit bodies related to their concentration in naturally and artificially polluted soil [26,27]. Sithole et al. [28] studied accumulation in mushrooms growing around three mining areas and reported that heavy metal contents can be significantly different. This confirms that both element concentration and soil chemistry influence the bioavailability of metals and their contents in fruit bodies. It seems that the enrichment of samples may be diverse, which is finally related to the potential toxicity of mushrooms and differing contents of essential inorganic elements [29].

Among the many industrial activities with negative environmental effects, the production of hazardous wastes poses serious environmental and social problems around the world. One group of such wastes is metal processing tailings. Flotation is a common mineral processing method used to upgrade copper sulfide ores where copper mineral particles are concentrated in froth, and associated gangue minerals are separated as tailings [30,31,32,33]. According to Zhai et al. [34], 60 million tons of Cu slag are generated annually worldwide during flotation and cause irreversible water and soil pollution. Finding an environmentally friendly remediation technology is crucial. There is potential for transforming tailing wastes into valuable products due to their considerable concentrations of many critical metals/metalloids. The recovery of elements and the use of the mineral residues in high- and low-value products can be very profitable from an industrial point of view (for producers of these pollutants and companies experienced in these methods). The amounts of generated wastes are so significant that a combination of several different approaches (reduce, reprocess, upcycle, downcycle) is needed [35]. One of the stages may be effective mycoremediation.

*Agaricus bisporus* is the most important commercially cultivated mushroom, contributing approximately 40–45% to world mushroom production [36,37]. Since it is so commonly cultivated, it would seem to be ideal for mycoremediation purposes. There are more and more reports of such use in the literature. Kryczyk et al. [38] presented in vitro cultures of *A. bisporus* demonstrating their remediation capacity for Cd and Pb from a supplemented medium. Kumar et al. [39] described an integrated approach for sustainable management of industrial wastewater and agricultural residues in *A. bisporus* production while minimizing the risks associated with their disposal. Ugya and Imam [40] described the effectiveness of *A. bisporus* in the remediation of refinery wastewater. The species showed high reduction efficiency for sulphate, phosphate, nitrate, alkalinity, electrical conductivity (EC), biological and chemical oxygen demands (BOD and COD), and heavy metals (Ag, Hg, Mn, Pb, and Zn). 

In view of the above, the aim of this study was to evaluate the mycoremediation ability of *A. bisporus*. The content of 51 elements in the mushroom fruit bodies growing on compost mixed with highly polluted flotation tailings in different quantities (1, 5, 10, 15, and 20% addition) enriched with flotation tailing was determined. The biomass of the collected fruit bodies was also assessed to estimate how polluted materials affect mushroom development. An experiment was performed to show the mineral composition of fruit bodies after the mycoremediation process.

## 2. Materials and Methods

### 2.1. Experimental Design

The substrate used for the experimental cultivation came from the commercial production of compost for mushroom growing (WRONA Company, Pszczyna, Poland). The compost was based on wheat straw, chicken manure, and gypsum and was prepared using a conventional method characteristic for phase II compost (fermentation and pasteurization). The compost was mixed with flotation tailings in quantities of 0 (control), 1 (FT_1_), 5 (FT_5_), 10 (FT_10_), 15 (FT_15,_), and 20% (FT_20_) by weight of the compost. Granulation [%] of flotation tailings was 11, 88, and 1 for clay, silt and sand, respectively. The pH of this component was 7.19, with an EC of 6.98 dS m^−1^. The characteristics of element concentration in flotation tailings are described in Table 1.

**Figure 1:**
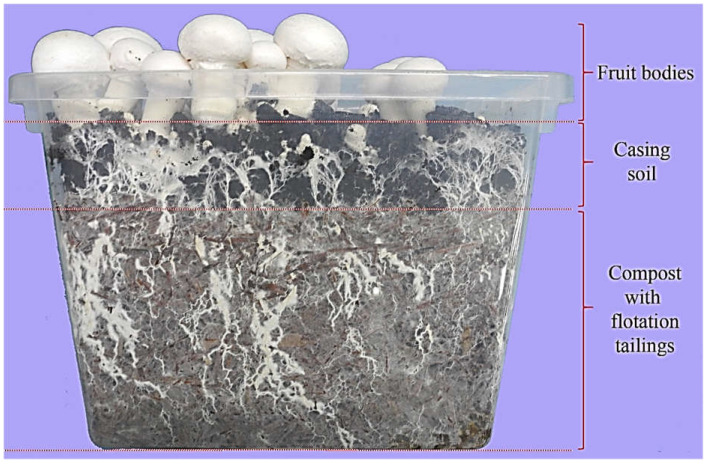
Preparation of experimental systems.

The compost was inoculated with commercial *A. bisporus* mycelium in 5% of the compost weight. The strain EuroMycel 58 was used. The mixtures were placed in plastic containers (15 × 18 × 14 cm) at 1 kg per container for each particular experimental system (Figure 1). The compost layer thickness was 8 cm. Eight containers for each treatment were prepared. Incubation was carried out in a growing chamber at a temperature of 24–25 °C and 85–90% of humidity. Once the compost was completely overgrown with mycelium, a 3.5 cm layer of casing soil was applied to the substrate surface. The moisture content of the casing soil was 75%. The casing soil came from the Wokas Company (Łosice, Poland) and was prepared based on sphagnum peat moss with chalk (pH 6.7). Incubation was continued until the mycelium overgrew the casing soil. When it appeared on the casing soil surface, the air temperature was lowered to 17–18 °C. The casing soil was watered to maintain constant moisture content. The growing chamber was aerated to keep CO_2_ concentration below 1500 ppm. The fruit bodies were collected fully developed but still completely closed. Individuals from the control and treatment FT_1_ were harvested simultaneously, whereas from the other experimental systems (FT_5_, FT_10_, FT_15,_ and FT_20_) 2, 3, 4, and 5 days later, respectively. The delay in the harvesting of fruiting bodies was due to the fact that increasing the amount of sludge (waste) addition delayed their setting.

Just two flushes of fruit bodies were produced and harvested. The interval between consecutive harvests was 8 days in each case. The yield included whole fruit bodies collected from 1 container, and the determined yield was a mean value calculated based on 8 containers belonging to the same treatment. None of the fruit bodies showed any signs of distortion or discoloration (Figure 1). The experiment was performed in May 2020. 

### 2.2. Analytical Procedure

All collected fruit bodies were carefully washed with distilled water from a Milli-Q Academic System (non-TOC) (Merck Millipore, Darmstadt, Germany) to remove substrate particles, and subsequently dried with paper towels and weighed. The mushrooms were then dried at 40 ± 1 °C to a constant weight in an electric oven (SLW 53 STD, Pol-Eko, Poland) and ground in a laboratory Cutting Boll Mill PM 200 (Retsch, Haan, Germany).

An accurately weighed 0.200–0.500 (±0.001) g of a sample was digested with 10 mL of concentrated nitric acid (HNO_3_; 65%; Sigma-Aldrich, Darmstadt, Germany) in closed Teflon containers in a microwave digestion system (Mars 6 Xpress, CEM, Matthews, NC, USA). Finally, the samples were filtered (Qualitative Filter Papers Whatman) and diluted to a volume of 15.0 mL with demineralized water (Direct-Q system, Millipore, USA). The inductively coupled plasma mass spectrometry system PlasmaQuant MS Q (Analytik Jena, Germany) was used to determine the following conditions: plasma gas flow 9.0 L min^−1^, nebulizer gas flow 1.05 L min^−1^, auxiliary gas flow 1.5 L min^−1^, radio frequency (RF) power 1.35 kW. The interferences were reduced using the integrated collision reaction cell (iCRC) working sequentially in three modes: with helium (He) as the collision gas, hydrogen (H) as the reaction gas, and without gas addition. The uncertainty for the analytical procedure, including sample preparation, was at the level of 20%. The detection limits were determined at the level of 0.001–0.010 mg kg^−1^ dry weight (DW) for all elements determined (3 times the standard deviation of the blank analysis (*n* = 10)). The accuracy was checked by analysis of the reference materials CRM 2709—soil; CRM S-1—loess soil; CRM 667—estuarine sediments; CRM 405—estuarine sediments; CRM NCSDC (73349)—bush branches and leaves, and the recovery (80–120%) was acceptable for most of the elements determined. For uncertified elements, recovery was defined using the standard addition method.

All the determined major and trace elements were divided into 6 groups, according to Kalač (2019):

(a) Major essential elements (MEEs): calcium (Ca), potassium (K), magnesium (Mg) and sodium (Na);
(b) Essential trace elements (ETEs): boron (B), cobalt (Co), copper (Cu), chromium (Cr), iron (Fe), manganese (Mn), nickel (Ni), selenium (Se) and zinc (Zn);
(c) Trace elements with detrimental health effects (TEWDHE): silver (Ag), arsenic (As), barium (Ba), cadmium (Cd), lead (Pb) and thallium (Tl);
(d) Rare earth elements (REEs): cerium (Ce), dysprosium (Dy), erbium (Er), europium (Eu), gadolinium (Gd), holmium (Ho), lanthanum (La), lutetium (Lu), neodymium (Nd), praseodymium (Pr), scandium (Sc), samarium (Sm), terbium (Tb), thulium (Tm), yttrium (Y) and ytterbium (Yb);
(e) Platinum group elements (PGEs): iridium (Ir), palladium (Pd), platinum (Pt), rhodium (Rh), ruthenium (Ru);
(f) Nutritionally non-essential elements (NNEs): aluminium (Al), gold (Au), bismuth (Bi), gallium (Ga), germanium (Ge), indium (In), lithium (Li), rhenium (Re), antimony (Sb), strontium (Sr), and tellurium (Te).

### 2.3. Statistical Analysis and Calculation

All statistical analyses were performed using the Agricole package (R). The analyses were performed in accordance with the procedure implemented in the R 3.6.1 environment [41]. To compare the content of determined elements in compost with different proportions of flotation tailings, a one-way ANOVA with Tukey’s HSD (statistically significant difference) post hoc: test was used. The same analysis was performed to compare the content of elements in fruit bodies growing in particular experimental treatments, separately for both flushes. This analysis used the Stat and Agricole package. A heatmap with a cluster analysis (implemented in the package Heatmaply) was performed to visualize multidimensional data separately for particular groups of elements and all elements jointly. An empirical normalization transformation brings data to the 0 to 1 scale and it allows comparison of variables of different scales, but it also keeps the shape of the distribution. A dendrogram was computed and reordered based on row and columns means. Additionally, to compare Ca, K, Mg, and Na contents together in compost with flotation tailings and fruit bodies from both flushes produced from particular experimental treatments, the rank-sum test was performed [42].

To estimate the efficiency of the element accumulation by mushrooms growing under particular treatments, the bioaccumulation factor (BAF) was calculated as a ratio of metal content in the whole fruit body dry matter to its concentration in substrate dry matter. 

## 3. Results

### 3.1. Fructification and Biomass Yield of A. bisporus 

**Figure 2:**
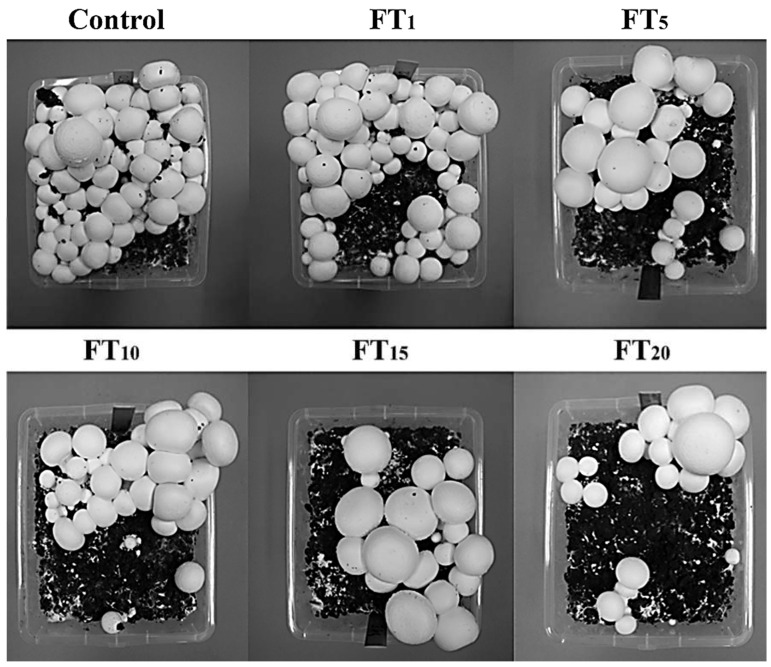
Macroscopic characteristics of *Agaricus bisporus* fruit bodies growing in particular experimental systems.

The fastest dynamic growth of *A. bisporus* fruit bodies was observed in the control treatment in both flushes. In contrast, the slowest growth was apparent for mushrooms growing under the FT_15_ and FT_20_ treatments. Generally, the size of fruit bodies growing under FT_5_ and FT_15_ treatments was greater than for the rest (including the control), which is partially visible in Figure 2. The same color characterized all the collected fruit bodies from both flushes. No negative symptoms as a result of the presence of toxic elements in flotation tailings were noted.

**Figure 3:**
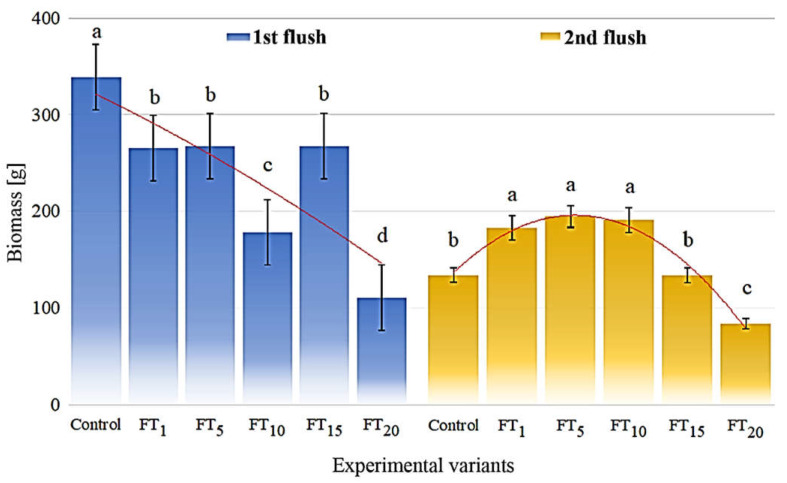
Biomass of *Agaricus bisporus* treated with particular flotation tailing addition; identical superscripts (a, b, c…) denote non-significant differences between means in columns determined in compost with flotation tailings and fruit bodies separately according to the post hoc Tukey’s HSD test.

Within the first flush, decreasing biomass yields of 339, 268, 267, 265, 178, and 111 g in the control, FT_5_, FT_15_, FT_1_, FT_10,_ and FT_20_, respectively, were recorded (Figure 3). The determined quadratic equation (y = −1.24x^2^ − 26.3x + 349; R^2^ = 0.6628) indicates a clear decrease in the biomass yield with an increasing proportion of flotation tailings in the substrate. The biomass of *A. bisporus* collected from the second flush increased from the control (134 g) to treatments FT_1_, FT_5,_ and FT_10_ with similar biomasses of 183, 194, and 191 g, respectively. The growth of fruit bodies under the FT_15_ system was related to the same biomass (134 g) as for the control, whereas the lowest biomass was observed for the FT_20_ (84.1 g). These changes are described by a quadratic equation (y = −13.7x^2^ + 84.3x + 65.8), which reflects (R^2^ = 0.9777) the fruit body response in relation to the increasing proportion of flotation tailings.

### 3.2. Content of Elements in Substrates

**Figure 4:**
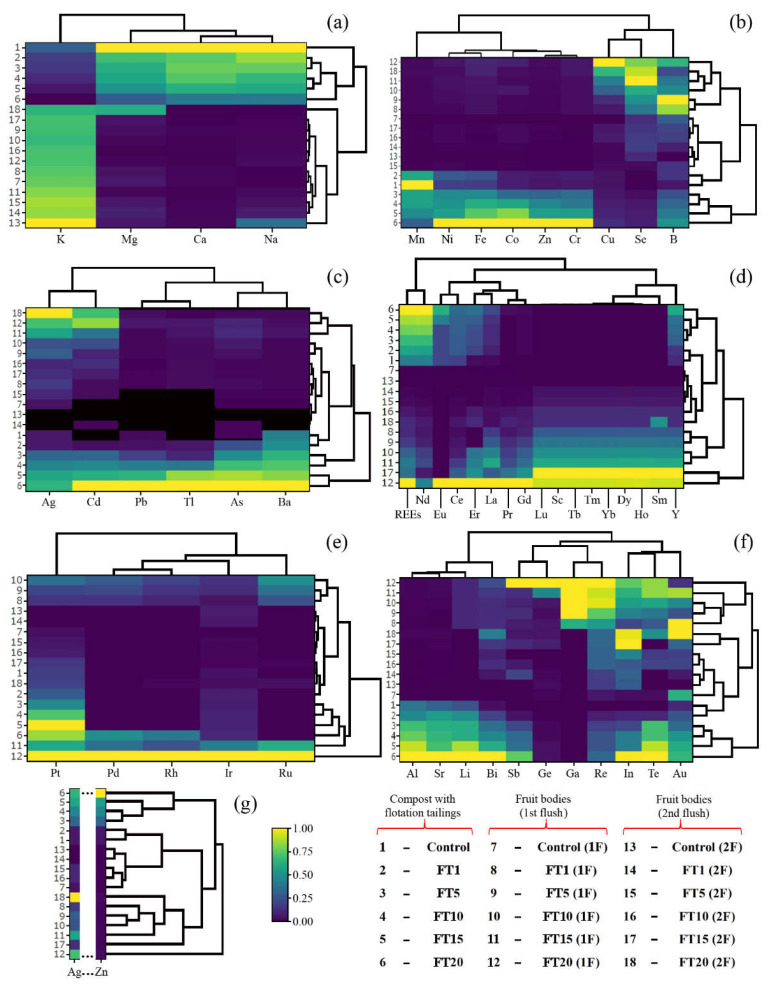
Correlation for compost with flotation tailings (1–6) and *Agaricus bisporus* collected from the 1st (7–12) and the 2nd flush (13–18) concerning the content of MEEs (**a**), ETEs (**b**), TEWDHE (**c**), REEs (**d**), PGEs (**e**), NNEs (**f**), and all elements jointly (**g**).

The addition of flotation tailings to the compost led to a lower mean content of MEEs from 6380 to 2550 mg kg^−1^ for Ca, from 3030 to 1560 mg kg^−1^ for K, from 726 to 282 mg kg^−1^ for Mg, and from 144 to 68.5 mg kg^−1^ for Na, for the control and FT_20_ (Table 2). Regarding the content of all MMEs in substrates, the highest similarities were observed between FT_1_ and FT_5_ or FT_10_ and FT_15_ (Figure 4a). 

The content of ETEs in compost with flotation tailings significantly increased for all the elements included in this group except Mn, where the highest content was determined in the control and the lowest in the FT_20_ system (55.6 and 17.0 mg kg^−1^, respectively) (Table 3). This observation confirms the heatmap, where the content of Mn in the control and FT1 belong to a separate group of objects (Figure 4b). The lowest and the highest mean contents of elements in the control and FT_20_ system were, in mg kg^−1^, as follows: 1.32 and 4.62 (B); 0.057 and 0.623 (Co); 0.287 and 3.66 (Cr); 6.35 and 14.7 (Cu); 29.5 and 158 (Fe); 0.405 and 2.12 (Ni); 0.027 and 0.239 (Se); and also 45.2 and 781 (Zn). The highest mean contents determined in the substrate from the FT_20_ system were: 359, 1093, 1275, 231, 536, 885, and 1728% of the content for the control, respectively, for B, Co, Cr, Cu, Fe, Ni, Se, and Zn. 

A significant increase in the mean TEWDHE content in the next treatment was also observed with the lowest value for the control (0.017, 1.55, 5.03, 0.036, 0.359, and 0.017 mg kg^−1^, respectively, for Ag, As, Ba, Cd, Pb, and Tl) and the highest for the FT_20_ system (0.107, 145, 11.6, 39.7, 15.8, and 1.87 mg kg^−1^, respectively) (Table 4). It is worth underlining that a high similarity was also observed between Ag and Cd, Pb and Tl, and As and Ba (Figure 4c). 

Mean ∑REEs ranged from 9.12 to 21.5 mg kg^−1^ for the control and FT_20_, respectively. The dominant REEs were Nd, from 7.92 to 18.7 mg kg^−1^ and Ce from 0.983 to 1.67 mg kg^−1^ (Table 5). This observation is confirmed by a heatmap, where, based on all experimental systems, the highest similarity is between ∑REEs and Nd (creating a separate group). At the same time, Ce, especially with Eu and Er, La, Pr, and Gd, create another group (Figure 4d). Moreover, the control and FT_1_ were similar, whereas the remaining systems created a new group of objects. 

Platinum was a dominant PGE with a mean content ranging from 0.323 to 1.42 mg kg^−1^, respectively, for the control and FT_20_ system (Table 6, Figure 4e). The addition of flotation tailings caused an increase in Pt content in the substrate. An increase in Ir content with the addition of flotation tailings was also observed from 0.150 to 0.323 mg kg^−1^, respectively, for the control and FT_20_ system. In the case of Pd and Rh, a significantly higher content of these elements was only observed in substrate FT_20_ compared with the other experimental systems.

For the NNEs, a significantly higher mean content of Al, Au, Bi, In, Li, Re, Sb, Sr, and Te was observed for the substrate in the FT_15_ and FT_20_ systems than for the control (Table 7). There were no significant differences in Ga and Ge content between the substrates in the control and particular treatments. All these observations are confirmed by a heatmap, where the control and FT_1_ systems create a separate group of objects and the highest contents, especially of Al, Bi, In, Li, Sr, and Te, are visible (Figure 4f). According to Figure 4g, where a heatmap for all detectable elements was prepared, the control and FT_1_ systems create a separate group, the same as the rest of the treatments used in this study. In contrast, there is generally an increased content of the studied elements in substrates with increased flotation tailings. 

### 3.3. Content of Elements in A. bisporus Fruit Bodies 

**Figure 5:**
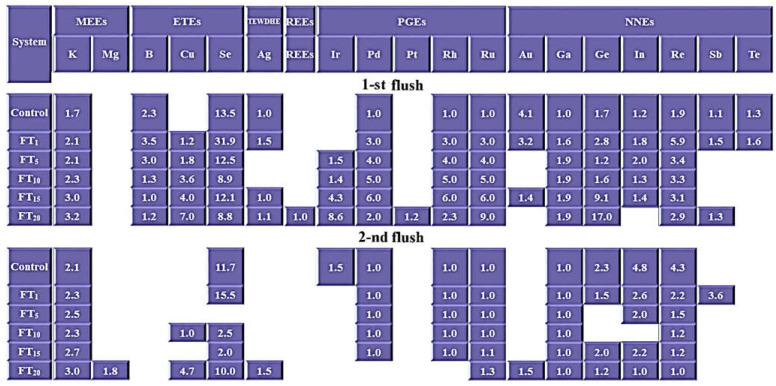
Bioaccumulation factor values for elements in fruit bodies collected from particular treatments.

The content of Ca significantly decreased in fruit bodies collected from the first (from 109 to 24.6 mg kg^−1^) and the second yield (from 100 to 24.6 mg kg^−1^) with an increase of flotation tailings in addition to the compost (Table 2). A similar trend was also observed for K and Mg (from the first yield only). The increased addition of flotation tailings to the compost did not generally cause significant changes in Mg content in fruit bodies from the second yield or Na in mushrooms from both yields. In this experiment, fruit bodies growing under a particular system were characterized by a high similarity between Ca and Na (Figure 4a). It is worth underlining that a heatmap with a separate object created for fruit bodies growing under the FT_20_ system from the second yield shows the effect of the lowest content of Ca, K, and Na (10.4, 4710, and 17.9 mg kg^−1^, respectively) and the highest content of Mg (494 mg kg^−1^). Only effective accumulation (BAF > 1) of K in *A. bisporus* from both yields and Mg in fruit bodies from the second yield under FT_20_ was observed (Figure 5).

The efficiency of ETE accumulation by mushroom species was highly diverse, which confirms a clear limited accumulation of Co, Cr, Fe, or Zn in mushrooms, especially from the second yield, and the opposite situation for Cu (Table 3). The highest mean content of ETEs was determined for mushrooms growing in substrates with higher additions of flotation tailings collected from the first (FT_10_ and/or FT_15_ and/or FT_20_) and the second yield (FT_20_), which confirms the heatmap where a separate group of objects was indicated for these treatments (Figure 4b). An apparent decrease of Mn content in the fruit bodies of *A. bisporus* was also confirmed by the heatmap, where this metal (similarly to B) is separated from the others (Figure 4b). BAF values higher than 1 were only observed for B, Cu, and Se in fruit bodies, mainly from the first yield (Figure 5). 

The content of TEWDHE in fruit bodies collected from the first yield was the highest under the FT_15_ and/or FT_20_ experimental systems, whereas from the second yield it was only under the FT_20_ system, except for Tl, where the highest content of this metal was recorded under FT_10_ and FT_15_ (0.062 and 0.058 mg kg^−1^, respectively) (Table 4). Despite this result, all mushrooms growing under the mentioned experimental systems created a separate group of objects, which undoubtedly shows similarities and differences concerning the other systems (Figure 4c). BAF > 1 was calculated only for Ag in *A. bisporus* bodies from the first (control, FT_1_, FT_15,_ and FT_20_) and the second (FT_20_) yield (Figure 5). 

∑REE content was the highest for *A. bisporus* growing under the FT_20_ system obtained from the first yield and FT_4_ from the second yield (21.6 and 7.88 mg kg^−1^, respectively) (Table 5), which confirms the graphical interpretation of the obtained results (a heatmap), where mushrooms growing on both these substrates are included in the same separate group of objects (Figure 4d). The dominant REEs were Nd and Ce in fruit bodies collected from both the first (1.14–8.12 and 0.142–4.94 mg kg^−1^, respectively) and the second yield (0.137–1.35 and 0.022–1.41 mg kg^−1^, respectively). The lower content of REEs in mushrooms growing under FT_20_ compared with FT_15_ from the second yield was probably the effect of the lower content of these elements in the substrate after the first yield and their generally limited accumulation in the second yield. The BAF calculated for ∑REEs in the first yield of *A. bisporus* growing only under FT_20_ was higher than 1 (Figure 5).

Effective accumulation of PGEs was determined, especially for Ir and Pt, whose highest mean content in fruit bodies under FT_20_ was 2.79 and 1.67 mg kg^−1^, respectively (first yield). In this case, the heatmap shows that mushrooms growing under this treatment create absolutely separate objects (Figure 4e). The efficiency of PGE accumulation by *A. bisporus* after the second yield was lower (Table 6). The BAF calculated for particular PGEs shows that values higher than 1 were observed, especially for the fruit bodies collected in the first yield growing under all the experimental systems (Pd, Rh, and Ru), FT_5_-FT_20_ (Ir), and FT_20_ (Pt) (Figure 5). In the second yield, BAF > 1 was also calculated, although it was usually an effect of a similar and low concentration of elements in the substrate and the content of these elements in mushrooms. 

The efficiency of NNE accumulation in fruit bodies was diverse, with generally the highest content found in mushrooms under the highest addition of flotation tailings, except for Au (1.14 mg kg^−1^ under FT_1_) and Sb (0.256 mg kg^−1^ under FT_1_) in bodies from the first and the second yield, respectively (Table 7). According to the heatmap, the high similarity concerning the content of all NNEs between mushrooms from the first yield was the same as between mushrooms from the second yield (Figure 4f). Effective accumulation (BAF > 1) of Ga, Ge, In, and Re was recorded for mushrooms growing under almost all experimental systems from both yields, whereas for the other NNEs only for mushrooms under the addition of selected flotation tailings (Figure 5).

Based on all determined elements in the collected fruit bodies growing under the control (both yields) and the FT_1_, FT_5,_ and FT_10_ systems from the second yield, a similarity was noted between them and their content in the substrates of the control FT_1_ (Figure 4g). On the other hand, *A. bisporus* growing under the remaining experimental systems enriched with flotation tailings (except for mushrooms under FT_20_ from the first yield) were characterized by a similar content of all determined elements, which are clearly shown as a separate group. The above-mentioned mushrooms growing under the FT_20_ system and collected from the first yield created a separate object characterized by the highest content of all studied elements jointly.

## 4. Discussion

Recently, there have been more and more reports on the possibility of using *A. bisporus* in remediation. In our experiment, five different quantities of flotation tailings were used to study the mycoremediation potential of this species. In the first flushes, a reduction in the yield was confirmed with the addition of flotation tailings. The literature confirms that supplementation of the substrate with metals/metalloids may reduce the growth dynamics of this species. Rzymski et al. [43] showed supplementation with Cu, Se, and Zn resulted in the biomass of fruiting bodies decreasing significantly at higher element addition (0.8 mM). Also, the addition of higher Hg concentrations (0.4 and 0.5 mM) to the growing medium reduced the growth of *A. bisporus* biomass [44].

The substrate in our experiment was richer in MEEs than mixtures of substrate and flotation tailings. The same tendency was confirmed in fruit bodies growing in experimental systems with their use. A different trend was observed concerning other groups of elements. The control substrate (compost) contained a significantly lower content of other elements than the substrate with flotation tailings. Because of the ability of *A. bisporus* to accumulate selected elements, the composition of waste affects the composition of its fruit (different amounts of waste addition resulting in different levels of elements in the substrate). Differences in the accumulation of heavy metals (Cd, Cr, and Pb) in *A. bisporus* fruiting bodies depending on the concentration of these elements in the growing medium were confirmed by Zhou et al. [45]. Nagy et al. [46] also confirmed that the maximum removal efficiencies from monocomponent aqueous solutions by *A. bisporus* for Cd and Zn took place at the highest concentrations of the substrate. The ability of *A. bisporus* to effectively accumulate selected elements shown in our study, is also well demonstrated by the results of our previous studies on supplementation during the cultivation of these mushrooms. Effective uptake of Cu, Se, and Zn from the enriched medium was confirmed by Rzymski et al. [43]. Supplementation with 0.6 mmol L^−1^ of Cu, Se, and Zn resulted in an over 3-fold, 2.5-fold, and 10-fold increase in their concentrations in fruiting bodies, respectively, whereas Rzymski et al. [44] demonstrated that Hg uptake increased in a concentration-dependent manner and exceeded 116 mg kg^−1^ in *A. bisporus* caps after 0.5 mM was added to the substrate.

BAF values may measure the mycoremediation efficiency of different mushroom species growing in polluted substrates and provide direct information about the potential of particular mushroom species to accumulate elements. This is crucial because this process has numerous dependent factors (environmental and genetic) [47], and is also the case for mushroom cultivation using different substrates as previously described [48]. In this study, effective accumulation was observed for selected elements only, which reflected the chosen additions of flotation tailings added to the compost. It suggests that *A. bisporus* application can be limited and/or used for the accumulation of selective elements only [13,49]. 

In general, mushroom fruit bodies collected after the mycoremediation process are waste that can be a substrate for the recovery of elements in the case of very high contents of especially precious elements [50]. The risk of contaminated food is genuine [51]. The problem of assessing the quality of fruit bodies (especially for significant and toxic trace elements) that could be consumed has been closely associated with a lack of appropriate legal regulations for many years [52,53]. However, even in countries where legislation exists, the regulations are limited to specific and usually toxic trace elements only [54,55]. The analysis and the further possibility of using the “product” from the mycoremediation of post-industrial wastes seems to be unlikely. Simultaneously, the risk of consuming contaminated fruit bodies is high, as may be shown in the study of Pająk et al. [56], who collected 10 mushroom species from polluted forest ecosystems.

## 5. Conclusions

The possibility of using mushroom fruiting bodies to decontaminate contaminated substrates effectively is an essential aspect of bioremediation due to their ability to accumulate elements effectively. The *A. bisporus* strain tested in this study effectively accumulated selected elements (Ag, Au, B, Cu, Ga, Ge, In, Ir, K, Mg, Pd, Pt, Re, Rh, Ru, Sb, Se, and Te), as evidenced by values of BAF > 1. Although this efficiency was not spectacular, to be able to recover elements in pure form, fundamentally enriched the fruiting bodies. The results of these studies indicate the potential for using *A. bisporus* fruiting bodies after the mycoremediation process in industry, although this must be preceded by larger-scale tests. This application seems to be the most favorable for media contaminated with selected elements, the absorption of which by fruiting bodies is the most efficient. Our study confirmed that the key to mycoremediation is determining the right fungal species to target a specific pollutant. However, due to the particularly effective accumulation of As and Cd, the post-flotation sediment subjected to this process should contain the lowest possible concentrations of both of these elements. Further research is necessary to determine the long-term potency of such a method. 

## Figures and Tables

**Table 1:** Concentration of elements [mg kg^−1^ dry weight] in flotation tailings used in experiment.

Elements Group	Element	Concentration	Elements Group	Element	Concentration
MEEs	Ca	12,700 ± 1510	REEs	Lu	0.197 ± 0.016
	K	7920 ± 231		Nd	95.6 ± 4.76
	Mg	2210 ± 187		Pr	0.814 ± 0.037
	Na	384 ± 24.6		Sc	0.980 ± 0.102
ETEs	B	27.9 ± 4.18		Sm	0.269 ± 0.035
	Co	4.87 ± 1.04		Tb	0.196 ± 0.021
	Cr	22.2 ± 2.29		Tm	0.446 ± 0.087
	Cu	238 ± 19.8		Y	1.08 ± 0.113
	Fe	884 ± 55.7		Yb	0.127 ± 0.036
	Mn	97.7 ± 10.1		**∑_REEs_**	123 ± 9.94
	Ni	28.3 ± 1.95			
	Se	3.77 ± 0.96	PGEs	Ir	1.96 ± 0.224
	Zn	4200 ± 1670		Pd	0.893 ± 0.055
TEWDHE	Ag	5.47 ± 1.02		Pt	11.7 ± 0.978
	As	818 ± 27.9		Rh	0.617 ± 0.042
	Ba	77.9 ± 6.64		Ru	0.135 ± 0.012
	Cd	188 ± 13.7	NNEs	Al	476 ± 35.2
	Pb	163 ± 11.0		Au	2.87 ± 0.138
	Tl	10.6 ± 1.14		Bi	1.43 ± 0.117
REEs	Ce	16.5 ± 2.01		Ga	0.201 ± 0.013
	Dy	1.23 ± 0.11		Ge	0.228 ± 0.024
	Er	2.64 ± 0.676		In	1.16 ± 0.112
	Eu	0.654 ± 0.024		Li	1.65 ± 0.097
	Gd	0.375 ± 0.016		Sb	7.65 ± 0.921
	Ho	0.127 ± 0.09		Sr	80.7 ± 5.34
	La	1.26 ± 0.076		Te	0.836 ± 0.101

MEEs—major essential elements; ETEs—essential trace elements; TEWDHE—trace elements with detrimental health effects; REEs—rare earth elements; PGEs—platinum group elements; NNEs—nutritionally non-essential elements.

**Table 2:** Content of major essential elements [mg kg^−1^ dry weight] in compost with flotation tailings before the experiment and fruit bodies from particular flushes.

System	Ca	K	Mg	Na
	Compost without/with Flotation Tailings			
Control	6380 ^a^	3030 ^a^	726 ^a^	144 ^a^
FT_1_	4720 ^b^	2420 ^ab^	536 ^b^	124 ^ab^
FT_5_	4930 ^b^	2350 ^ab^	495 ^b^	114 ^ab^
FT_10_	4700 ^b^	2130 ^bc^	474 ^b^	101 ^b^
FT_15_	4020 ^b^	1820 ^bc^	428 ^bc^	94.0 ^bc^
FT_20_	2550 ^c^	1560 ^c^	282 ^c^	68.5 ^bc^
System	1st flush			
Control	109 ^a^	5280 ^b^	148 ^a^	17.5 ^c^
FT_1_	92.6 ^b^	5150 ^bc^	140 ^ab^	18.0 ^bc^
FT_5_	83.1 ^b^	4900 ^d^	133 ^ab^	18.3 ^abc^
FT_10_	51.9 ^c^	4800 ^e^	122 ^b^	19.2 ^abc^
FT_15_	43.5 ^c^	5540 ^a^	120 ^b^	20.5 ^ab^
FT_20_	24.6 ^d^	5010 ^c^	119 ^b^	20.9 ^a^
System	2nd flush			
Control	100 ^a^	6420 ^a^	148 ^b^	62.8 ^a^
FT_1_	82.0 ^b^	5660 ^b^	165 ^b^	26.3 ^b^
FT_5_	63.9 ^c^	5770 ^ab^	150 ^b^	22.7 ^b^
FT_10_	48.2 ^d^	4990 ^c^	111 ^b^	19.2 ^b^
FT_15_	40.6 ^e^	4970 ^c^	142 ^b^	18.6 ^b^
FT_20_	10.4 ^f^	4710 ^c^	494 ^a^	17.9 ^b^

*n* = 3; identical superscripts (a, b, c…) denote non-significant differences between means in columns determined in compost with flotation tailings and fruit bodies separately according to the post hoc Tukey’s HSD test.

**Table 3:** Content of essential trace elements [mg kg^−1^ dry weight] in compost with flotation tailings before the experiment and fruit bodies from particular flushes.

System	B	Co	Cr	Cu	Fe	Mn	Ni	Se	Zn
	Compost without/with Flotation Tailings								
Control	1.32 ^e^	0.057 ^e^	0.287 ^d^	6.35 ^d^	29.5 ^e^	55.6 ^a^	0.405 ^c^	0.027 ^c^	45.2 ^d^
FT_1_	2.03 ^d^	0.060 ^e^	0.351 ^d^	6.66 ^d^	42.8 ^e^	33.3 ^b^	0.611 ^c^	0.033 ^c^	54.0 ^d^
FT_5_	2.73 ^c^	0.217 ^d^	1.31 ^c^	7.72 ^cd^	76.6 ^d^	32.3 ^b^	1.01 ^b^	0.108 ^b^	255 ^c^
FT_10_	3.34 ^bc^	0.367 ^c^	1.50 ^c^	9.26 ^bc^	99.0 ^c^	31.7 ^b^	1.08 ^b^	0.185 ^a^	364 ^b^
FT_15_	3.91 ^b^	0.513 ^b^	2.07 ^b^	11.0 ^b^	117 ^b^	26.6 ^cb^	1.19 ^b^	0.216 ^a^	441 ^b^
FT_20_	4.62 ^a^	0.623 ^a^	3.66 ^a^	14.7 ^a^	158 ^a^	17.0 ^c^	2.12 ^a^	0.239 ^a^	781 ^a^
System	1st flush								
Control	3.05 ^e^	0.010 ^c^	0.061 ^c^	1.64 ^e^	2.04 ^d^	0.40 ^d^	0.017 ^c^	0.367 ^f^	4.17 ^d^
FT_1_	7.19 ^de^	0.019 ^bc^	0.180 ^b^	8.05 ^de^	8.56 ^c^	1.26 ^c^	0.048 ^bc^	1.05 ^e^	14.6 ^c^
FT_5_	8.30 ^d^	0.019 ^bc^	0.176 ^b^	14.1 ^d^	9.00 ^bc^	1.31 ^bc^	0.080 ^ab^	1.35 ^d^	14.8 ^c^
FT_10_	4.43 ^c^	0.050 ^bc^	0.175 ^b^	32.9 ^c^	9.98 ^bc^	1.40 ^abc^	0.087 ^ab^	1.65 ^c^	16.1 ^bc^
FT_15_	3.81 ^b^	0.025 ^ab^	0.184 ^b^	44.2 ^b^	10.3 ^b^	1.46 ^ab^	0.105 ^ab^	2.62 ^b^	17.9 ^b^
FT_20_	5.73 ^a^	0.062 ^a^	0.254 ^a^	103 ^a^	17.1 ^a^	1.52 ^a^	0.124 ^a^	2.11 ^a^	26.4 ^a^
System	2nd flush								
Control	0.892 ^e^	0.010 ^b^	0.064 ^b^	3.21 ^b^	2.48 ^b^	0.37 ^b^	0.014 ^b^	0.318 ^b^	6.63 ^d^
FT_1_	1.53 ^d^	0.013 ^ab^	0.064 ^b^	3.35 ^b^	2.84 ^b^	0.49 ^b^	0.026 ^ab^	0.511 ^b^	6.95 ^cd^
FT_5_	1.96 ^c^	0.013 ^ab^	0.061 ^b^	4.31 ^b^	2.20 ^b^	0.52 ^b^	0.037 ^ab^	0.092 ^b^	7.30 ^cd^
FT_10_	2.27 ^b^	0.023 ^ab^	0.062 ^b^	8.94 ^b^	2.30 ^b^	0.67 ^b^	0.053 ^ab^	0.466 ^b^	9.07 ^bc^
FT_15_	2.36 ^ab^	0.023 ^ab^	0.058 ^b^	7.52 ^b^	3.54 ^b^	0.64 ^b^	0.053 ^ab^	0.432 ^b^	9.86 ^b^
FT_20_	2.62 ^a^	0.030 ^a^	0.273 ^a^	68.8 ^a^	16.9 ^a^	1.79 ^a^	0.075 ^a^	2.38 ^a^	20.8 ^a^

*n* = 3; identical superscripts (a, b, c…) denote non-significant differences between means in columns determined in compost with flotation tailings and fruit bodies separately according to the post hoc Tukey’s HSD test

**Table 4:** Content of trace elements with detrimental health effects [mg kg^−1^ dry weight] in compost with flotation tailings before the experiment and fruit bodies from particular flushes.

System	Ag	As	Ba	Cd	Pb	Tl
	Compost without/with Flotation Tailings					
Control	0.017 ^d^	1.55 ^f^	5.03 ^e^	0.036 ^e^	0.359 ^e^	0.017 ^d^
FT_1_	0.023 ^d^	35.2 ^e^	5.76 ^de^	1.51 ^e^	0.675 ^e^	0.108 ^d^
FT_5_	0.060 ^c^	71.2 ^d^	7.52 ^cd^	13.1 ^d^	3.07 ^d^	0.189 ^d^
FT_10_	0.077 ^bc^	100 ^c^	8.42 ^bc^	18.7 ^c^	6.55 ^c^	0.743 ^c^
FT_15_	0.100 ^ab^	126 ^b^	9.48 ^b^	25.0 ^b^	10.2 ^b^	1.58 ^b^
FT_20_	0.107 ^a^	145 ^a^	11.6 ^a^	39.7 ^a^	15.8 ^a^	1.87 ^a^
System	1st flush					
Control	0.017 ^b^	0.330 ^d^	0.141 ^d^	0.019 ^f^	0.047 ^d^	0.010 ^c^
FT_1_	0.035 ^b^	4.22 ^c^	0.285 ^c^	1.14 ^e^	0.171 ^c^	0.064 ^b^
FT_5_	0.055 ^b^	5.76 ^c^	0.337 ^c^	3.87 ^d^	0.264 ^bc^	0.054 ^b^
FT_10_	0.049 ^b^	9.85 ^b^	0.273 ^c^	9.46 ^c^	0.272 ^bc^	0.057 ^b^
FT_15_	0.100 ^a^	16.4 ^a^	0.707 ^a^	15.2 ^b^	0.328 ^b^	0.105 ^a^
FT_20_	0.118 ^a^	14.6 ^a^	0.511 ^b^	33.3 ^a^	1.03 ^a^	0.118 ^a^
System	2nd flush					
Control	0.010 ^b^	0.137 ^d^	0.128 ^d^	0.021 ^c^	0.032 ^b^	0.011 ^b^
FT_1_	0.010 ^b^	1.19 ^c^	0.155 ^d^	0.320 ^c^	0.032 ^b^	0.011 ^b^
FT_5_	0.027 ^b^	2.04 ^b^	0.214 ^c^	1.22 ^bc^	0.092 ^b^	0.010 ^b^
FT_10_	0.027 ^b^	1.98 ^b^	0.288 ^b^	5.09 ^b^	0.062 ^b^	0.062 ^a^
FT_15_	0.030 ^b^	2.22 ^b^	0.318 ^b^	3.02 ^bc^	0.173 ^b^	0.058 ^a^
FT_20_	0.163 ^a^	3.02 ^a^	0.390 ^a^	27.9 ^a^	0.546 ^a^	0.013 ^b^

*n* = 3; identical superscripts (a, b, c…) denote non-significant differences between means in columns determined in compost with flotation tailings and fruit bodies separately according to the post hoc Tukey’s HSD test.

**Table 5:** Content of rare earth elements [mg kg^−1^ dry weight] in compost with flotation tailings before the experiment and fruit bodies from particular flushes.

System	Ce	Dy	Er	Eu	Gd	Ho	La	Lu	Nd	Pr	Sc	Sm	Tb	Tm	Y	Yb	∑_REEs_
	Compost without/with Flotation Tailings																
Control	0.983 ^b^	0.010 ^a^	0.041 ^d^	0.010 ^a^	0.013 ^b^	0.010 ^a^	0.027 ^c^	0.010 ^a^	7.92 ^d^	0.010 ^b^	0.010 ^a^	0.010 ^a^	0.010 ^a^	0.010 ^a^	0.040 ^c^	0.010 ^a^	9.12 ^d^
FT_1_	1.38 ^ab^	0.010 ^a^	0.293 ^c^	0.010 ^a^	0.027 ^ab^	0.010 ^a^	0.093 ^b^	0.010 ^a^	10.6 ^c^	0.010 ^b^	0.010 ^a^	0.010 ^a^	0.010 ^a^	0.010 ^a^	0.053 ^bc^	0.010 ^a^	12.6 ^c^
FT_5_	1.42 ^ab^	0.010 ^a^	0.337 ^cb^	0.013 ^a^	0.030 ^ab^	0.010 ^a^	0.123 ^b^	0.010 ^a^	12.5 ^c^	0.010 ^b^	0.010 ^a^	0.010 ^a^	0.010 ^a^	0.010 ^a^	0.090 ^bc^	0.010 ^a^	14.6 ^c^
FT_10_	1.46 ^a^	0.010 ^a^	0.340 ^cb^	0.010 ^a^	0.033 ^ab^	0.010 ^a^	0.117 ^b^	0.010 ^a^	14.8 ^b^	0.010 ^b^	0.010 ^a^	0.010 ^a^	0.010 ^a^	0.010 ^a^	0.097 ^bc^	0.010 ^a^	17.0 ^b^
FT_15_	1.53 ^a^	0.010 ^a^	0.423 ^ab^	0.020 ^a^	0.033 ^ab^	0.010 ^a^	0.143 ^b^	0.010 ^a^	16.5 ^ab^	0.010 ^b^	0.010 ^a^	0.010 ^a^	0.010 ^a^	0.010 ^a^	0.107 ^b^	0.010 ^a^	18.8 ^b^
FT_20_	1.67 ^a^	0.010 ^a^	0.537 ^a^	0.027 ^a^	0.043 ^a^	0.010 ^a^	0.213 ^a^	0.010 ^a^	18.7 ^a^	0.093 ^a^	0.010 ^a^	0.010 ^a^	0.010 ^a^	0.010 ^a^	0.173 ^a^	0.010 ^a^	21.5 ^a^
System	1st flush																
Control	0.010 ^c^	0.010 ^f^	bDL	bDL	0.013 ^c^	0.010 ^f^	0.013 ^f^	0.010 ^f^	0.077 ^c^	0.013 ^b^	0.010 ^f^	0.010 ^f^	0.011 ^f^	0.010 ^f^	0.010 ^f^	0.013 ^f^	0.221 ^f^
FT_1_	0.142 ^bc^	0.074 ^e^	bDL	bDL	0.142 ^bc^	0.088 ^e^	0.170 ^e^	0.084 ^e^	1.14 ^c^	0.142 ^b^	0.085 ^e^	0.096 ^e^	0.089 ^e^	0.086 ^e^	0.081 ^e^	0.082 ^e^	2.50 ^e^
FT_5_	0.265 ^bc^	0.119 ^d^	bDL	bDL	0.265 ^bc^	0.114 ^d^	0.265 ^d^	0.109 ^d^	1.59 ^c^	0.303 ^b^	0.108 ^d^	0.120 ^d^	0.123 ^d^	0.105 ^d^	0.117 ^d^	0.111 ^d^	3.71 ^d^
FT_10_	0.378 ^bc^	0.142 ^c^	0.568 ^b^	bDL	0.426 ^ba^	0.142 ^c^	0.426 ^c^	0.142 ^c^	3.31 ^b^	0.426 ^b^	0.142 ^c^	0.142 ^c^	0.142 ^c^	0.142 ^c^	0.142 ^c^	0.142 ^c^	6.81 ^c^
FT_15_	0.851 ^b^	0.180 ^b^	0.624 ^a^	bDL	0.511 ^b^	0.175 ^b^	0.568 ^b^	0.160 ^b^	4.20 ^b^	0.568 ^b^	0.164 ^b^	0.170 ^b^	0.165 ^b^	0.176 ^b^	0.170 ^b^	0.172 ^b^	8.85 ^b^
FT_20_	4.94 ^a^	0.255 ^a^	1.46 ^b^	0.047	1.45 ^a^	0.252 ^a^	0.937 ^a^	0.255 ^a^	8.12 ^a^	2.384 ^a^	0.275 ^a^	0.256 ^a^	0.265 ^a^	0.238 ^a^	0.255 ^a^	0.244 ^a^	21.6 ^a^
System	2n d flush																
Control	0.010 ^b^	0.010 ^e^	bDL ^c^	bDL	0.010 ^b^	0.010 ^b^	0.010 ^b^	0.010 ^b^	0.013 ^d^	0.010 ^b^	0.010 ^b^	0.010 ^c^	0.010 ^b^	0.010 ^b^	0.010 ^b^	0.010 ^b^	0.144 ^e^
FT_1_	0.022 ^b^	0.022 ^d^	bDL ^c^	bDL	0.021 ^b^	0.023 ^b^	0.026 ^b^	0.023 ^b^	0.137 ^cd^	0.023 ^bc^	0.023 ^b^	0.027 ^c^	0.020 ^b^	0.025 ^b^	0.019 ^b^	0.024 ^b^	0.433 ^de^
FT_5_	0.064 ^b^	0.025 ^c^	bDL ^c^	bDL	0.023 ^b^	0.031 ^b^	0.028 ^b^	0.029 ^b^	0.292 ^c^	0.024 ^bc^	0.022 ^b^	0.032 ^c^	0.037 ^b^	0.018 ^b^	0.027 ^b^	0.030 ^b^	0.685 ^d^
FT_10_	0.098 ^b^	0.042 ^b^	0.112 ^b^	bDL	0.098 ^b^	0.044 ^b^	0.043 ^b^	0.042 ^b^	0.619 ^b^	0.127 ^bc^	0.046 ^b^	0.042 ^c^	0.040 ^b^	0.043 ^b^	0.044 ^b^	0.043 ^b^	1.49 ^c^
FT_15_	1.41 ^a^	0.273 ^a^	0.718 ^a^	bDL	0.736 ^a^	0.272 ^a^	0.279 ^a^	0.276 ^a^	1.35 ^a^	0.915 ^a^	0.277 ^a^	0.279 ^a^	0.276 ^a^	0.278 ^a^	0.274 ^a^	0.276 ^a^	7.88 ^a^
FT_20_	0.289 ^b^	0.046 ^b^	0.159 ^b^	bDL	0.116 ^b^	0.043 ^b^	0.040 ^b^	0.033 ^b^	1.31 ^a^	0.231 ^b^	0.048 ^b^	0.144 ^b^	0.050 ^b^	0.043 ^b^	0.049 ^b^	0.045 ^b^	2.65 ^b^

*n* = 3; identical superscripts (a, b, c…) denote non-significant differences between means in columns determined in compost with flotation tailings and fruit bodies separately according to the post hoc Tukey’s HSD test; bDL—below the detection limit.

**Table 6:** Content of platinum group elements [mg kg^−1^ dry weight] in compost with flotation tailings before the experiment and fruit bodies from particular flushes.

System	Ir	Pd	Pt	Rh	Ru
	Compost without/with Flotation Tailings				
Control	0.150 ^c^	0.010 ^b^	0.323 ^e^	0.010 ^b^	0.010 ^a^
FT_1_	0.237 ^b^	0.010 ^b^	0.486 ^e^	0.010 ^b^	0.010 ^a^
FT_5_	0.247 ^b^	0.010 ^b^	0.803 ^d^	0.010 ^b^	0.010 ^a^
FT_10_	0.283 ^ab^	0.010 ^b^	1.22 ^c^	0.010 ^b^	0.010 ^a^
FT_15_	0.277 ^ab^	0.010 ^b^	1.65 ^a^	0.010 ^b^	0.010 ^a^
FT_20_	0.323 ^a^	0.077 ^a^	1.42 ^b^	0.090 ^a^	0.010 ^a^
System	1st flush				
Control	0.023 ^d^	0.010 ^b^	0.087 ^e^	0.010 ^b^	0.010 ^f^
FT_1_	0.160 ^cd^	0.030 ^b^	0.278 ^d^	0.030 ^b^	0.030 ^e^
FT_5_	0.360 ^cd^	0.040 ^b^	0.366 ^d^	0.040 ^b^	0.040 ^d^
FT_10_	0.407 ^c^	0.050 ^b^	0.612 ^c^	0.050 ^b^	0.050 ^c^
FT_15_	1.18 ^b^	0.060 ^b^	1.02 ^b^	0.060 ^b^	0.060 ^b^
FT_20_	2.79 ^a^	0.150 ^a^	1.67 ^a^	0.210 ^a^	0.090 ^a^
System	2nd flush				
Control	0.227 ^a^	0.010 ^a^	0.032 ^c^	0.010 ^a^	0.010 ^a^
FT_1_	0.197 ^a^	0.010 ^a^	0.032 ^c^	0.010 ^a^	0.010 ^a^
FT_5_	0.073 ^bc^	0.010 ^a^	0.122 ^bc^	0.010 ^a^	0.010 ^a^
FT_10_	0.073 ^bc^	0.010 ^a^	0.155 ^b^	0.010 ^a^	0.010 ^a^
FT_15_	0.050 ^c^	0.010 ^a^	0.288 ^a^	0.010 ^a^	0.011 ^a^
FT_20_	0.110 ^b^	0.010 ^a^	0.351 ^a^	0.010 ^a^	0.010 ^a^

*n* = 3; identical superscripts (a, b, c…) denote non-significant differences between means in columns determined in compost with flotation tailings and fruit bodies separately according to the post hoc Tukey’s HSD test.

**Table 7:** Content of nutritionally non-essential elements [mg kg^−1^ dry weight] in compost with flotation tailings before the experiment and fruit bodies from particular flushes.

System	Al	Au	Bi	Ga	Ge	In	Li	Re	Sb	Sr	Te
	Compost without/with Flotation Tailings										
Control	26.7 ^f^	0.187 ^d^	0.024 ^e^	0.010 ^a^	0.010 ^a^	0.013 ^d^	0.033 ^c^	0.013 ^c^	0.012 ^e^	3.43 ^c^	0.010 ^b^
FT_1_	32.0 ^e^	0.357 ^c^	0.045 ^e^	0.010 ^a^	0.013 ^a^	0.027 ^cd^	0.047 ^bc^	0.027 ^c^	0.072 ^d^	5.90 ^b^	0.023 ^b^
FT_5_	47.0 ^d^	0.427 ^c^	0.079 ^d^	0.010 ^a^	0.020 ^a^	0.048 ^c^	0.060 ^abc^	0.064 ^b^	0.267 ^c^	6.80 ^b^	0.077 ^a^
FT_10_	52.5 ^c^	0.633 ^b^	0.104 ^c^	0.010 ^a^	0.023 ^a^	0.089 ^b^	0.063 ^abc^	0.082 ^ab^	0.433 ^b^	7.10 ^b^	0.077 ^a^
FT_15_	62.5 ^b^	0.727 ^ab^	0.134 ^b^	0.010 ^a^	0.027 ^a^	0.093 ^b^	0.077 ^ab^	0.084 ^ab^	0.452 ^b^	7.59 ^b^	0.100 ^a^
FT_20_	68.5 ^a^	0.777 ^a^	0.198 ^a^	0.011 ^a^	0.030 ^a^	0.198 ^a^	0.087 ^a^	0.099 ^a^	0.936 ^a^	10.9 ^a^	0.110 ^a^
System	1st flush										
Control	0.203 ^d^	0.757 ^c^	0.013 ^c^	0.010 ^c^	0.017 ^c^	0.016 ^c^	0.010 ^a^	0.026 ^d^	0.013 ^b^	0.013 ^d^	0.013 ^c^
FT_1_	0.642 ^c^	1.14 ^a^	0.037 ^b^	0.016 ^b^	0.037 ^c^	0.049 ^bc^	0.019 ^a^	0.161 ^c^	0.105 ^b^	0.037 ^cd^	0.037 ^bc^
FT_5_	0.779 ^bc^	0.343 ^e^	0.036 ^b^	0.019 ^a^	0.025 ^c^	0.095 ^ab^	0.019 ^a^	0.218 ^b^	0.161 ^b^	0.057 ^c^	0.050 ^abc^
FT_10_	0.779 ^bc^	0.580 ^d^	0.036 ^b^	0.019 ^a^	0.037 ^c^	0.116 ^a^	0.019 ^a^	0.273 ^a^	0.125 ^b^	0.109 ^b^	0.069 ^ab^
FT_15_	0.931 ^a^	1.01 ^b^	0.044 ^a^	0.019 ^a^	0.243 ^b^	0.133 ^a^	0.019 ^a^	0.264 ^a^	0.175 ^b^	0.228 ^a^	0.094 ^a^
FT_20_	1.035 ^a^	0.231 ^f^	0.057 ^a^	0.019 ^a^	0.511 ^a^	0.155 ^a^	0.020 ^a^	0.285 ^a^	1.240 ^a^	0.264 ^a^	0.094 ^a^
System	2n d flush										
Control	0.313 ^b^	0.073 ^d^	0.013 ^c^	0.010 ^a^	0.023 ^b^	0.064 ^b^	0.010 ^a^	0.058 ^b^	0.011 ^d^	0.011 ^c^	0.010 ^a^
FT_1_	0.343 ^b^	0.147 ^d^	0.024 ^bc^	0.010 ^a^	0.020 ^b^	0.070 ^b^	0.010 ^a^	0.061 ^b^	0.256 ^a^	0.032 ^c^	0.010 ^a^
FT_5_	0.363 ^b^	0.277 ^c^	0.030 ^bc^	0.010 ^a^	0.010 ^c^	0.096 ^b^	0.010 ^a^	0.095 ^a^	0.010 ^d^	0.061 ^bc^	0.027 ^a^
FT_10_	0.407 ^b^	0.293 ^c^	0.037 ^b^	0.010 ^a^	0.010 ^c^	0.062 ^b^	0.010 ^a^	0.096 ^a^	0.093 ^c^	0.031 ^c^	0.030 ^a^
FT_15_	0.417 ^b^	0.420 ^b^	0.043 ^b^	0.012 ^a^	0.053 ^a^	0.202 ^a^	0.011 ^a^	0.099 ^a^	0.144 ^b^	0.086 ^b^	0.010 ^a^
FT_20_	1.32 ^a^	1.15 ^a^	0.092 ^a^	0.012 ^a^	0.047 ^a^	0.195 ^a^	0.013 ^a^	0.098 ^a^	0.078 ^c^	0.195 ^a^	0.059 ^a^

*n* = 3; identical superscripts (a, b, c…) denote non-significant differences between means in columns determined in compost with flotation tailings and fruit bodies separately according to the post hoc Tukey’s HSD test.

## Data Availability

Not applicable.
